# PlasticDB: a database of microorganisms and proteins linked to plastic biodegradation

**DOI:** 10.1093/database/baac008

**Published:** 2022-03-09

**Authors:** Victor Gambarini, Olga Pantos, Joanne M Kingsbury, Louise Weaver, Kim M Handley, Gavin Lear

**Affiliations:** School of Biological Sciences, University of Auckland, 3a Symonds Street, Auckland 1010, New Zealand; The Institute of Environmental Science and Research, 27 Creyke Road, Ilam, Christchurch 8041, New Zealand; The Institute of Environmental Science and Research, 27 Creyke Road, Ilam, Christchurch 8041, New Zealand; The Institute of Environmental Science and Research, 27 Creyke Road, Ilam, Christchurch 8041, New Zealand; School of Biological Sciences, University of Auckland, 3a Symonds Street, Auckland 1010, New Zealand; School of Biological Sciences, University of Auckland, 3a Symonds Street, Auckland 1010, New Zealand

## Abstract

The number of publications reporting putative plastic-degrading microbes and proteins is continuously increasing, necessitating the compilation of these data and the development of tools to facilitate their analysis. We developed the PlasticDB web application to address this need, which comprises a database of microorganisms and proteins reported to biodegrade plastics. Associated metadata, such as the techniques utilized to assess biodegradation, the environmental source of microbial isolate and presumed thermophilic traits are also reported. Proteins in the database are categorized according to the plastic type they are reported to degrade. Each protein structure has been predicted *in silico* and can be visualized or downloaded for further investigation. In addition to standard database functionalities, such as searching, filtering and retrieving database records, we implemented several analytical tools that accept inputs, including gene, genome, metagenome, transcriptomes, metatranscriptomes and taxa table data. Users can now analyze their datasets for the presence of putative plastic-degrading species and potential plastic-degrading proteins and pathways from those species.

Database URL:http://plasticdb.org.

## Introduction

Plastics are polymeric materials that have been widely manufactured for an extensive range of industrial and household products in the past 80 years (1). Depending on the polymer type, plastics possess many desirable properties, including typically low production costs, being lightweight but with good impact resistance, being relatively inert, available in transparent to opaque forms and typically having good resistance to chemical as well as biological degradation (2). Such properties led global plastic production to increase exponentially (3) from 2 million tonnes in 1950 to 400 million tonnes in 2015, with production expected to double in the next 20 years (4).

The widespread production and use of plastics combined with their long-term durability and poor waste management have caused progressive environmental accumulation. Plastics can damage marine life, harming species at the base of the food chain to the largest animals on Earth (5). For example, the growth and photosynthetic capacity of the most abundant photosynthetic organism on Earth, the marine cyanobacteria *Prochlorococcus*, is demonstrated to be impacted by plastic leachate (6). Large filter-feeding animals such as the baleen whales can possess levels of microplastics four orders of magnitude greater than expected from measurements of microplastics in local coastal surface waters. This suggests that trophic transfer may be occurring and highlights the potential widespread exposure of marine organisms to microplastics (7).

Researchers have attempted to employ physical, chemical and biological methods to degrade waste plastic. Physical and chemical degradation typically requires high temperature or pressure or chemicals that may be expensive or themselves harmful, restricting their application while sometimes can generate degradation products that may also cause damage to the environment (8). Biological plastic degradation is often considered a more environmentally friendly method, receiving considerable attention from the scientific community. It occurs when microorganisms use their enzymatic apparatus to break down polymers into smaller molecules and monomers. These may be used as carbon and energy sources and are ultimately mineralized by microorganisms, being converted into carbon dioxide, water, methane and other compounds (9). Biological processes can usually be performed under mild environmental conditions (such as lower temperatures, pressures and pH levels), circumventing the utilization and production of dangerous chemicals (10) and thereby possibly reducing processing costs. However, for many plastics, biological processes for polymer degradation remain to be demonstrated commercially. With this in mind, scientists have been exploring the potential of microorganisms to biodegrade plastics (11, 12).

The first reported microbial degrader of a synthetic plastic polymer was described in 1974 when the ubiquitous and generalist fungus *Aureobasidium pullulans* was demonstrated to biodegrade polycaprolactone (PCL) (13). In 1977, Tokiwa and Suzuki (14) described a fungus from the *Penicillium* genus that also degraded PCL, with Benedict et al. (15) isolating four microorganisms able to degrade PCL in 1983. At around the same time, Shimao et al. (16) isolated a bacterium from the *Pseudomonas* genus that could degrade polyvinyl alcohol (PVA). After a slow beginning, research into microbial plastic degradation started to escalate; by 1990, another five studies had been published (17–21). In the following decade, the growth was even more significant; from 1991 to 2000, a further 66 studies on microbial degradation of plastics were published, elevating the number of species reported to biodegrade plastics from 22 species in 1990 to 129 species in 2000. By 2020, over 400 articles described plastic degradation by over 400 microbial species.

We previously compiled information on microbial species and proteins associated with reports of plastic biodegradation, demonstrating that presumed plastic-degrading traits are widely dispersed across the microbial tree of life. Our dataset includes more than a hundred proteins identified to break down plastics, noting that it is not always possible from these to distinguish if enzymes are capable of degrading the virgin polymer, as opposed to plastic contaminants and physicochemical degradation products. More than 16 000 putative plastic-degradation orthologs of these genes reside in the genomes of 6000 microbial species, most of which are not currently reported as being plastic degraders. These species belong to twelve different microbial phyla, yet to date, just seven phyla include taxa for which microbial plastic degradation is reported (22).

Two major efforts to gather and organize current literature in microbial plastic degradation include the PMBD (23) published in 2019 and the work of Gambarini et al. (22) published at the beginning of 2021. The PMBD database has an excellent collection of microorganisms and proteins with around 390 species and 79 proteins. It also has two tools to align and predict potential plastic degradation proteins. However, the database has not been updated since (as of December 2021) its release. It does not include information on the structure of proteins presumed capable of plastic degradation or the capacity to analyze genome, metagenome or taxa table data. Gambarini et al. (22) expanded considerably the number of putative plastic-degrading species and proteins captured from the literature and explored their phylogenetic and global distribution. However, this literature database lacked a dedicated web server and any tools for users to analyze their own data.

Here, we provide a revised and updated dataset of microorganisms and proteins reported to degrade plastics in combination with a web application that allows database searches and data visualization. We also developed several tools for potential users to investigate multiple aspects of plastic biodegradation using their own datasets. These tools can be used to identify microorganisms and proteins that may be involved in plastic biodegradation, compare the genetic potential for plastic biodegradation across datasets, analyze plastic biodegradation pathways and explore the structural data of all proteins reported in the literature.

## Methods

### Implementation

We implemented the PlasticDB web application using Python (version 3.7) and Flask (version 1.1.2) on a server running the Ubuntu (version 20.01) operating system. The front-end design was created using the HTML and CSS languages and the Bootstrap framework. Interactive graphs were created using the Python library Plotly. Additionally, we used AlphaFold2 (24) to predict the structure of all proteins within the database; iCn3D (25) was integrated to visualize three-dimensional protein structures.

### Data collection

To explore the current literature for evidence of microbial plastic degradation, we gathered peer-reviewed publications through two methods: (i) obtaining all publications released up to August 2021 using the Web of Science platform with the following keywords: [plastic* AND *degradation AND (bacter* OR fung* OR archaea*)]; (ii) collecting all other information that we knew to exist, such as studies that were described in published reviews, and all taxa found in the PMBD database (23) that met our criteria for inclusion. Data shown in this publication cover reports published up to August 2021; however, the database is updated regularly.

Our literature search was conducted to obtain a general overview of microorganisms reported to degrade plastics. However, our keywords may have missed some microorganisms, plastics and proteins; similar searches using terms such as *eukaryot* and diatom* returned no results. To fit our criteria and therefore be added to our database, the publications had to include: (i) evidence of plastic degradation by an isolated microorganism; (ii) multiple methods to assess plastic biodegradation; weight loss alone was not considered as evidence of biodegradation because this technique does not differentiate between the biodegradation of additives or polymers. In addition, the mere isolation of microorganisms from plastic surfaces, plastic-contaminated environments, or media were not treated as proof of biodegradation.

### Analysis tools

To facilitate the use of our database, we created and incorporated several analysis tools into the web application. These tools take as input several common data types (Table 1) and identify microorganisms and proteins that may be linked to plastic biodegradation. Sequence alignments are performed using the DIAMOND algorithm (26) on protein sequences of putative plastic-degrading enzymes in our database, which were previously reported in the literature. Users can specify e-value and identity cutoffs for the DIAMOND search to adjust the stringency of the search criteria and select protein or nucleotide input data.

**Table 1.** Inputs and parameters that can be specified for each of PlasticDB’s tools

Tool	Input	Parameters
Annotate Gene	A FASTA or FASTQ file containing one sequence in nucleotide or amino acid format. Files can contain assembled or raw reads and be uncompressed or compressed using gzip.	File type, BLAST type, e-value, percent identity and microorganism type.
Annotate Genome	A FASTA or FASTQ file containing multiple sequences in nucleotide or amino acid format. Files can contain assembled or raw reads and be uncompressed or compressed using gzip.	File type, BLAST type, e-value, percent identity and microorganism type.
Annotate Taxa Table	A taxa table file containing at least genus and species names.	Field separator, genus column, species column.
Compare Genomes	Multiple FASTA or FASTQ files containing multiple sequences in nucleotide or amino acid format. Files can contain assembled or raw reads and be uncompressed or compressed using gzip.	File type, BLAST type, e-value and percent identity.
Pathway Analysis	A FASTA or FASTQ file containing multiple sequences in nucleotide or amino acid format. Files can contain assembled or raw reads and be uncompressed or compressed using gzip.	File type, BLAST type, e-value and percent identity.

Since plastic polymers are usually too large to penetrate the cell membrane, microorganisms need to secrete biodegradative enzymes into the environment to break down plastics. Therefore, a critical piece of information for assessing the potential for plastic degradation by proteins is whether these proteins are secreted or not. For this reason, our pipeline implements a search for signal peptides (i.e. mechanisms for extracellular protein secretion) using the Signalp 5.0 software (27). If the protein is predicted to have a signal peptide it may, however, be retained inside the cell or have transmembrane helices and therefore be retained in the cell membrane.

We developed an Annotate Taxa Table tool to deal with amplicon sequence data, such as the outputs of QIIME (28) and DADA2 (29) pipelines. The tool compares the species or genus present in a submitted taxa table to species or genera in the PlasticDB database that are reported to biodegrade plastic. Users can specify the column numbers where genus and species information are located in their input data and which delimiter character is used to separate fields. The ETE Toolkit (30) algorithm searches the PlasticDB database, allowing for synonym handling in species names. The nomenclature system is kept updated using the most updated version of the NCBI taxonomic database (31).

To visualize plastic degradation data in the context of biological pathways, we developed a Pathway Analysis tool. The only complete pathway for plastic biodegradation described to date is found in the bacterium *Ideonella sakaiensis* (32); as a result, it is the only pathway currently provided in our database. As new reports are released, additional pathways will be added. PathVisio 3 (33) was used to draw the pathway, and annotations are marked in the pathway using a custom Python algorithm based on the Python package gpml2svg (pypi.org/project/gpml2svg/).

## Results and discussion

### Database statistics

**Figure 1.**
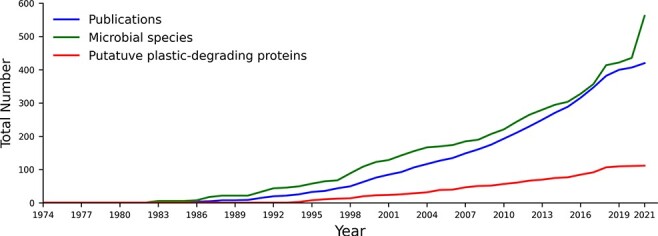
The cumulative number of publications, microbial species and proteins reported to degrade plastics between 1974 and August 2021. All included data fit our criteria for putative plastics degradation, as outlined in the methods.

PlasticDB contains information on microorganisms and proteins reported in the scientific literature linked to plastic biodegradation. It presently includes data from 421 scientific publications, representing 562 microbial species. These species make up 1462 records since each different plastic and each different reference accounts for a separate record (i.e. one microbial taxon may be linked to the degradation of multiple plastic types). We also identified 111 proteins reported to degrade plastics (Figure 1).

**Figure 2.**
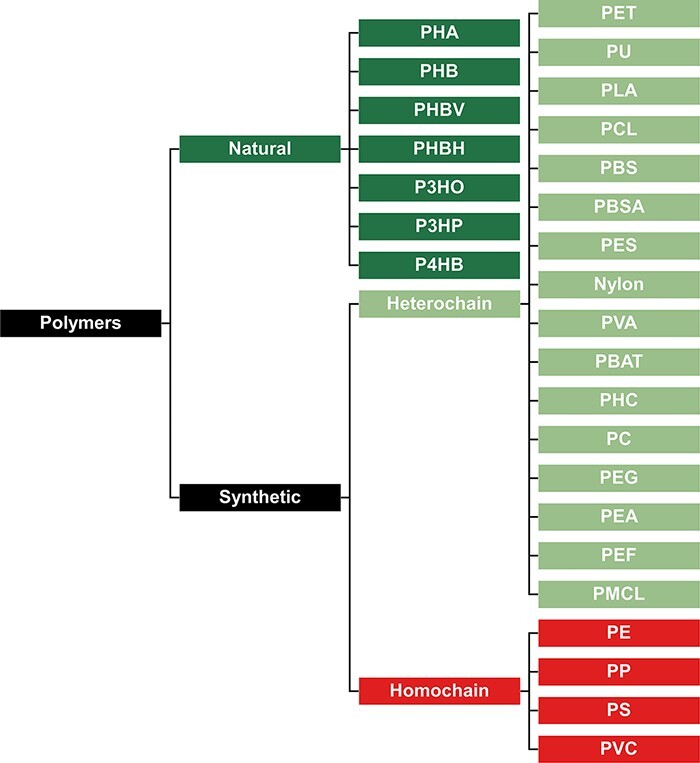
Classification of polymers to represent their presumed biodegradation potential. Natural polymers are more biodegradable, synthetic heterochain polymers have an intermediate biodegradability, and synthetic homochain polymers are the least biodegradable.

To best interpret the results within our database, it is important to classify all plastic types in groups that best represent their biodegradation potential. Our first classification divides all plastic types into natural and synthetic polymers (Figure 2). Natural polymers are comprised of polymers resulting from a process that has taken place in nature, irrespective of the process that is now used to mass-produce the polymer. On the other hand, synthetic polymers are manufactured materials that have never occurred in nature before. This classification is important for assessing biodegradation potential since natural polymers typically biodegrade faster than synthetic polymers, as microorganisms have already had time to evolve enzymatic systems to break them down (34–36). Synthetic polymers can be classified as ‘heterochain’ and ‘homochain’ polymers. Heterochain polymers have heteroatoms such as oxygen or nitrogen in their polymer backbones, while homochain polymers have extensive inert C–C backbone structures that are devoid of functional groups. These functional groups make heterochain polymers substantially more susceptible to enzymatic hydrolysis, and consequently, they have far greater biodegradation potential (37).

**Figure 3.**
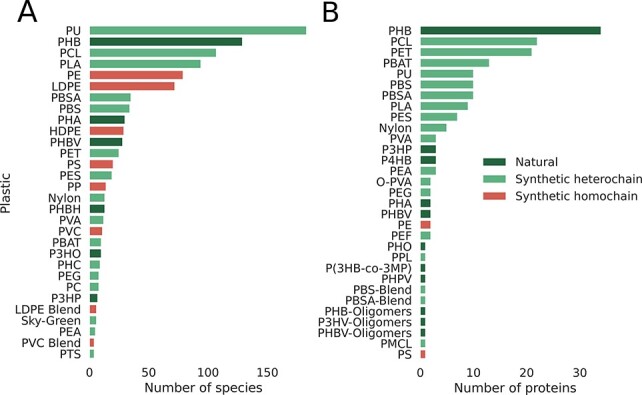
Database statistics per plastic, emphasizing the smaller number of reports for synthetic homochain polymers (those composed of only C–C backbones). A) Number of microbial species reported to degrade plastics. B) Number of proteins reported to break down plastics categorized by plastic type.

The classification shown in Figure 2 directly impacts our database since reports for the biodegradation of natural polymers and synthetic heterochain polymers are extensively documented in the literature. On the other hand, evidence is weak for the microbial degradation of synthetic homochain polymers. As Lear et al. (38) highlight, most studies lack clear confirmation of microbial degradation of high-weight polymer versus losses of plastic additives or physicochemical degradation products. This difference in biodegradation potential is depicted in Figure 3. When we look at the number of species reported to biodegrade all three categories of plastics (Figure 3A), natural and heterochain polymers have a far greater number of reported taxa. Polyethylene is the only homochain polymer that has a comparable number of reported species, but most studies lack strong evidence of polymer biodegradation (39). This absence of strong biodegradation becomes even more apparent when we compare the number of proteins reported to degrade plastics from each of these categories (Figure 3B). The synthetic homochain polymers polyethylene and polystyrene have just two and one protein reported, respectively. On the contrary, the natural polymer polyhydroxybutyrate (PHB) has 37 reported putative degradative proteins, while the synthetic heterochain polymer polycaprolactone has 22.

### Retrieving taxon and protein data

**Figure 4.**
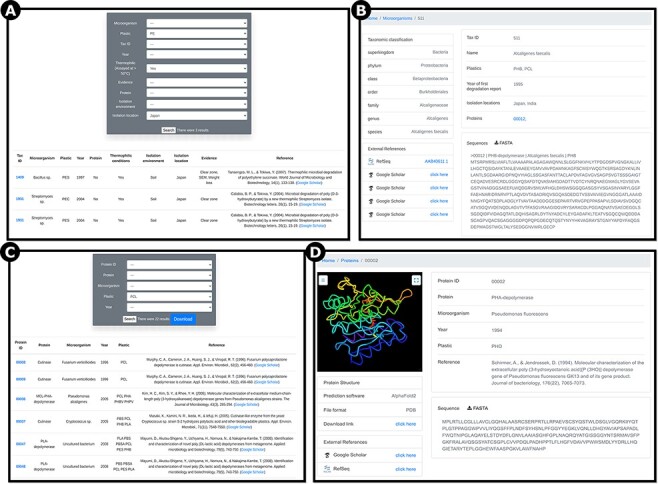
Screenshots of example pages showing information on reported plastic-degrading microorganisms and proteins. A) ‘Microorganisms’ page showing search results filtered for polyethylene (PE), thermophilic organisms, and isolation location in Japan; B) ‘Microorganisms’ page outputs showing *Alcaligenes faecalis,* a bacterium reported to degrade PHB and PCL; C) ‘Proteins’ page showing 22 results for the plastic type polycaprolactone (PCL). D) ‘Proteins’ page showing Pseudomonas fluorescens PHA-depolymerase, reported to break down polyhydroxyoctanoate (PHO; RefSeq ID AAA64538.1). Users can visualize the predicted protein structure, download the prediction in PDB file format and download the sequence in FASTA file format.

To allow users to easily search and filter all of the information on PlasticDB, we created two main pages, one for microorganisms and one for proteins. When using the ‘microorganisms’ page (plasticdb.org/microorganisms), users can apply filters to search only records that match specific criteria, such as species name, plastic type, tax id, publication year, confirmation of thermophilic attributes, laboratory evidence for plastic degradation, protein type, isolation environment and isolation location (Figure 4A). By clicking on the tax id number of any database record, the user is taken to a page that shows all information specific to that microorganism, with all plastic types it is reported to biodegrade, all proteins that have been identified as breaking down those plastics, the respective references and all additional details as provided in the taxon’s biodegradation report (Figure 4B).

There are currently (as of August 2021) 111 proteins in the database (plasticdb.org/proteins). Users can apply filters to search only records that match specific criteria, such as protein ID, protein type, microorganism, plastic and the publication year (Figure 4C), and click on the tax id number of any database record to obtain a more detailed biodegradation report (Figure 4D). On this page, users can also visualize the AlphaFold2 predicted protein structure. Finally, users can download a FASTA file with the protein sequence; the predicted protein structure can also be downloaded in the protein data bank (or PDB) file format.

### Analysis tools

**Figure 5.**
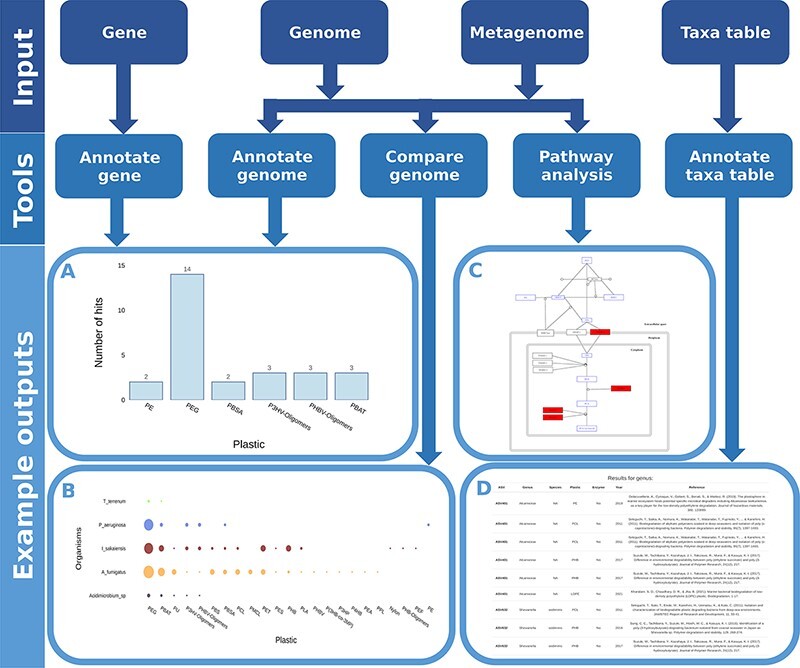
Flow chart showing all possible inputs, respective tools and example outputs for each data type. A) The *Ideonella sakaiensis* genome was used as an input for the ‘Annotate Genome’ tool, showing the number of input proteins that matched database proteins, grouped by plastic type. B) Comparison of plastic biodegradation potential for the genomes of *Ideonella sakaiensis,*  *Agaricus bisporus,*  *P. aeruginosa* and *Aspergillus fumigatus.* C) The *P. aeruginosa* genome was also used as the input for the ‘Pathway Analysis’ tool. Open blue rectangles represent substrates, solid red rectangles represent proteins in the pathway present in the *P. aeruginosa* genome, and open black rectangles represent proteins not present in the genome. D) An example output table generated by the ‘Annotate Taxa Table’ tool using amplicon sequencing data as the input. All figures are available at higher resolution in the supplementary material.

We developed and integrated various analytical tools into the web application. These tools accept numerous common data types as input (Figure 5) and identify microorganisms and proteins associated with plastic biodegradation.

#### ‘Annotate Gene’ and ‘Annotate Genome’ tools

Uncovering genes and enzymes responsible for the biodegradation of plastics is a key goal of many studies in the field; however, the number of publications reporting degradation-conferring genes and enzymes represents just a small fraction of biodegradation reports. Most studies identify just the microorganisms and not the genes and enzymes. To help researchers fulfill this need, we developed two tools; the first annotates a single gene (plasticdb.org/annotategene), the second annotates full genomes (plasticdb.org/annotategenome). To use these tools, users just need to upload a FASTA file with a nucleotide or protein sequence to search these against all sequences in our database. The outputs of both tools are very similar; example outputs for the ‘Annotate Gene’ tool are given in Supplementary Table S1 and Supplementary Figure S1.

**Figure 6.**
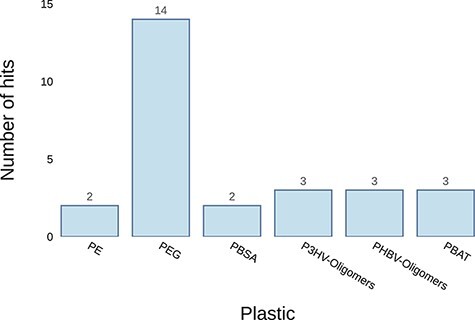
Example graph output from the ‘Annotate Genome’ tool. It plots the number of results returned for putative plastic-degrading proteins per plastic type. The input datum was the genome of Pseudomonas aeruginosa.

The ‘Annotate Genome’ tool can be useful, for instance, when a microorganism with presumed plastic-degrading capabilities has been isolated and its genome sequenced. Using this tool, researchers can identify genes similar to those previously reported to confer plastic biodegradation. The examples in Table 2 and Figure 6 show the results of uploading the genome of *Pseudomonas aeruginosa* to the server. The results show that it has at least two potential genes associated with PE biodegradation, WP_003083349.1 and WP_003102475.1. Both genes had matches for an alkane-hydroxylase isolated from *Pseudomonas* sp. by Yoon et al. (40) and an alkane-monooxygenase isolated from *Paenibacillus* sp. by Bardají et al. (41). Another useful piece of information that can guide efforts to identify the genes responsible for their plastic-degrading ability is whether the proteins translated from these genes are secreted or not; therefore, prediction of protein secretion is included in the results table.

**Table 2.** Example output from the ‘Annotate Genome’ tool showing the top ten hits found in the database. The input datum was the genome of *Pseudomonas aeruginosa*

Query Sequence	DB Hit ID Number	Percent Identity	E-value	Enzyme Type	Species	Plastic	Secreted
WP_003083349.1	00061	91.7	1.6e-72	Alkane-hydroxylase	*Pseudomonas* sp.	PE	No
WP_003083349.1	00104	51.1	7.4e-39	Alkane-monooxygenase	*Paenibacillus* sp.	PE	No
WP_003084198.1	00035	33.3	2.1e-69	PEG-aldehyde-dehydrogenase	*Streptomyces* sp.	PEG	No
WP_003095318.1	00056	43.5	1.8e-66	Lipase	*Burkholderia cepacia*	PBSA	Yes
WP_003095318.1	00052	41.2	1.2e-41	Lipase	*Pseudomonas aeruginosa*	PBSA	Yes
WP_003098285.1	00035	36.7	2.6e-85	PEG-aldehyde-dehydrogenase	*Streptomyces* sp.	PEG	No
WP_003101377.1	00035	35.6	2.0e-70	PEG-aldehyde-dehydrogenase	*Streptomyces* sp.	PEG	No
WP_003102147.1	00032	34.2	1.2e-70	PEG-dehydrogenase	*Sphingomonas macrogoltabidus*	PEG	Yes
WP_003102475.1	00061	75.9	6.2e-59	Alkane-hydroxylase	*Pseudomonas* sp.	PE	No
WP_003102475.1	00104	45.0	7.9e-32	Alkane-monooxygenase	*Paenibacillus* sp.	PE	No

#### ‘Annotate Taxa Table’ tool

Several studies on the taxonomic composition of plastic-associated microorganisms have been published recently. To better understand the microbial dynamics of these ‘plastisphere’ communities, the identification of taxa with the potential to biodegrade the plastics they are colonizing is of benefit. For these studies, we have developed the tool ‘Annotate Taxa Table’ (plasticdb.org/annotatelist). This tool’s input is a taxa table, usually an output of amplicon sequencing pipelines, such as QIIME (28) and DADA2 (29). The output is a table showing all species found in the PlasticDB database that have previously been reported to degrade plastics, the year the report has been published, the reference, and if there are proteins that break down plastics isolated from these taxa (Table 3).

**Table 3.** Example output from the ‘Annotate Taxa Table’ tool showing the top ten lines of a list of species in the user dataset that have already been reported as potential plastic degraders in the literature. The input datum was a taxa table generated from amplicon sequencing data through the DADA2 pipeline

ASV	Genus	Species	Plastic	Enzyme	Year	Reference
ASV632	*Shewanella*	sediminis	PCL	No	2011	Sekiguchi, T., Sato, T., Enoki, M., Kanehiro, H., Uematsu, K., & Kato, C. (2011). JAMSTEC Report of Research and Development, 11, 33–41.
ASV632	*Shewanella*	sediminis	PHB	No	2016	Sung, C. C., Tachibana, Y., Suzuki, M., Hsieh, W. C., & Kasuya, K. I. (2016). Polymer degradation and stability, 129, 268–274.
ASV632	*Shewanell* *a*	sediminis	PHB	No	2017	Suzuki, M., Tachibana, Y., Kazahaya, J. I., Takizawa, R., Muroi, F., & Kasuya, K. I. (2017). Journal of Polymer Research, 24 (12), 217.
ASV703	*Alteromonas*	genovensis	PHBH	No	2019	Kato, C., Honma, A., Sato, S., Okura, T., Fukuda, R., & Nogi, Y. (2019). High Pressure Research, 1–10.
ASV801	*Pseudoalteromonas*	denitrificans	PHBH	No	2018	Morohoshi, T., Ogata, K., Okura, T., & Sato, S. (2018). Microbes and Environments, ME17052.
ASV819	*Vibrio*	splendidus	PCL	PET-hydrolase	2018	Danso, D., Schmeisser, C., Chow, J., Zimmermann, W., Wei, R., Leggewie, C., … & Streit, W. R. (2018). Appl. Environ. Microbiol., 84 (8), e02773–17.
ASV819	*Vibrio*	splendidus	PET	PET-hydrolase	2018	Danso, D., Schmeisser, C., Chow, J., Zimmermann, W., Wei, R., Leggewie, C., … & Streit, W. R. (2018). Appl. Environ. Microbiol., 84 (8), e02773–17.
ASV819	*Vibrio*	splendidus	PVA Blend	No	2014	Raghul, S. S., Bhat, S. G., Chandrasekaran, M., Francis, V., & Thachil, E. T. (2014). International Journal of Environmental Science and Technology, 11 (7), 1827–1834.
ASV819	*Vibrio*	splendidus	LLDPE Blend	No	2015	Raghul, S. S., Bhat, S. G., Chandrasekaran, M., Francis, V., & Thachil, E. T. (2014). International Journal of Environmental Science and Technology, 11 (7), 1827–1834.
ASV819	*Vibrio*	splendidus	Nylon	No	2007	Sudhakar, M., Priyadarshini, C., Doble, M., Murthy, P. S., & Venkatesan, R. (2007). International Biodeterioration & Biodegradation, 60 (3), 144–151.

#### ‘Compare Genomes’ tool

**Figure 7.**
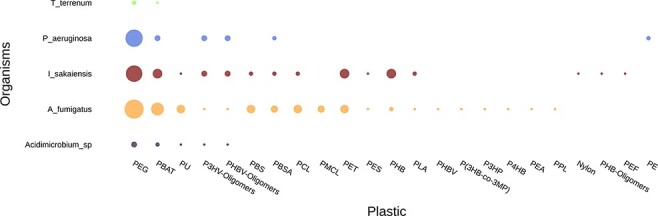
Example graph output from the ‘Compare Genome’ tool. The tool plots the number of hits for putative plastic-degrading proteins per plastic type for each dataset. The size of the dots represents the number of hits found in each genome for each plastic. The input data were the genomes of *Thermobaculum terrenum,*  *Pseudomonas aeruginosa,*  *Ideonella sakaiensis,*  *Aspergillus fumigatus* and *Acidimicrobium* sp.

Comparing the plastic biodegradation potential of different organisms or communities is very important when bioprospecting microbes and enzymes. This is an emerging area of research and just a few studies have been published so far; for instance, Bryant et al. (42) and Pinnell and Turner (43) investigated the metagenomes of communities inhabiting plastic debris. The ‘Compare Genomes’ tool annotates genomes or metagenomes and generates graphs and tables comparing all entries regarding their plastic biodegradation potential. Figure 7 and Table 4 are example outputs where five genomes are compared: *Thermobaculum terrenum, Pseudomonas aeruginosa, Ideonella sakaiensis, Aspergillus fumigatus* and *Acidimicrobium sp*.

#### ‘Pathway Analysis’ tool

**Figure 8.**
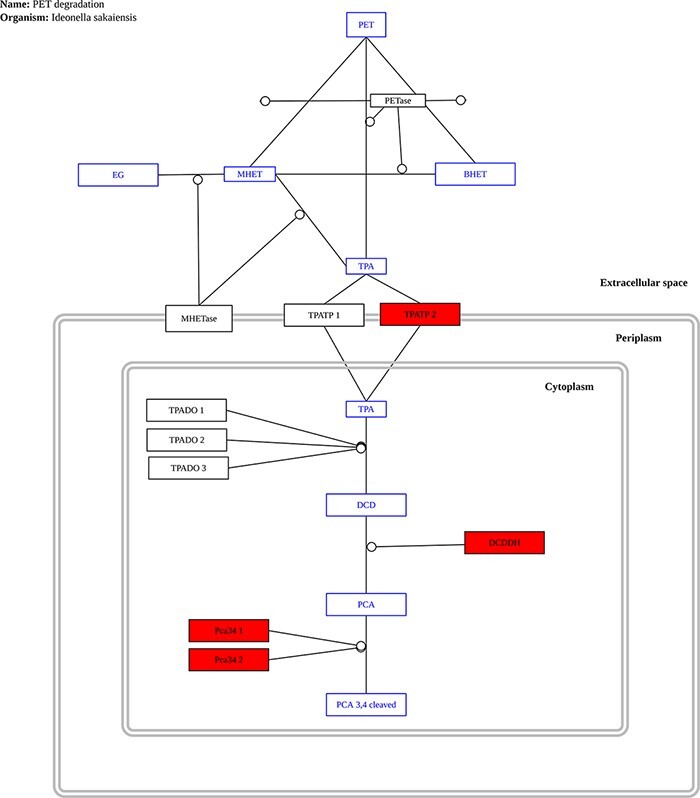
Example graph output from the ‘Pathway Analysis’ tool. Black rectangles represent proteins, blue rectangles represent substrates and red rectangles represent proteins present in the genome or metagenome being investigated, in this case, the genome of the bacterium P. aeruginosa.

The only complete pathway for plastic biodegradation described to date is that found in the bacterium *Ideonella sakaiensis* (32); as a result, it is the only pathway currently provided in our database. As new reports are released, additional pathways will be added. The user only needs to upload a file with all predicted proteins in a genome or metagenome to use this tool. The proteins are compared to the proteins found in *I. sakaiensis* that make up the PET biodegradation pathway and any matches in the pathway are highlighted. Figure 8 shows an example output for the genome of *P. aeruginosa*.

#### ‘Interactive Phylogenetic Tree’ tool

**Figure 9.**
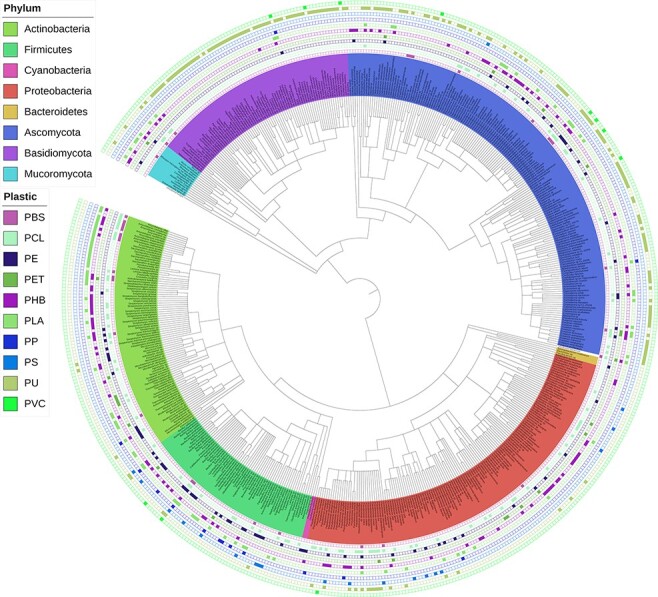
Interactive phylogenetic tree showing all microbes in the database and their respective plastic degradation reports. This figure is updated regularly with new reports of microbial degradation. Available at plasticdb.org/interactive_tree.

An interactive phylogenetic tree, published and periodically updated by Gambarini et al. (22), is integrated into our web application (Figure 9). Researchers can use this tool to obtain a global view of the current knowledge on species reported to biodegrade plastics and, more specifically, identify phylogenetic relationships among degraders of specific plastic types. For instance, it appears that polyurethane (PU) biodegradation has been more extensively investigated in fungi. At the same time, a cluster of phylogenetically related species within the family Pseudonocardiaceae are reported to be capable of polylactic acid (PLA) degradation.

## Conclusion

**Table 4.** Example output from the Compare Genomes tool shows the number of database hits grouped by plastic type for each genome

Samples	Nylon	PEG	PES	P3HV-Oligomers	PMCL	PHB	PBAT	PEA	PBSA	P4HB	P3HP	P(3HB-co-3MP)	PLA	PPL	PHBV	PHBV-Oligomers	PU	PET	PBS	PEF	PHB-Oligomers	PCL	PE
I_sakaiensis	1	13	1	3	0	6	6	0	2	0	0	0	2	0	0	3	1	6	2	1	1	2	0
Acidimicrobium_sp	0	3	0	1	0	0	2	0	0	0	0	0	0	0	0	1	1	0	0	0	0	0	0
T_terrenum	0	2	0	0	0	0	1	0	0	0	0	0	0	0	0	0	0	0	0	0	0	0	0
A_fumigatus	0	16	1	1	4	2	9	1	4	1	1	1	1	1	1	1	5	5	5	0	0	5	0
P_aeruginosa	0	14	0	3	0	0	3	0	2	0	0	0	0	0	0	3	0	0	0	0	0	0	2

Our freely accessible web application for the analysis of microbial plastic biodegradation data comprises the largest library of microbes and proteins reported to break down a wide range of plastics. Users can utilize the web server’s analytic tools to investigate multiple aspects of plastic biodegradation in their datasets, including identification of microorganisms and proteins potentially involved in plastic biodegradation, comparison of plastic biodegradation potential among different datasets, presence of complete or partial pathways for plastic biodegradation and analysis of structural data for all proteins reported in the literature. The PlasticDB web application can generate graphs and tables for visualization and interpretation, making it a valuable resource for all researchers investigating microbial plastic degradation. As a result, our application benefits this emerging research field by enhancing our understanding of the genetic variety and development of microbial plastic-degrading traits.

## Supplementary Material

baac008_Supp
