# Supplementary material for: PlasticDB: a database of microorganisms and proteins linked to plastic biodegradation
Source: Database (Oxford). 2022 Mar 9;2022:baac008. doi: 10.1093/database/baac008 (PMC9216477; doi:10.1093/database/baac008)
Supplement: baac008_Supp [file baac008_supp.zip › Supplementary_data_v6.docx]

**Supplementary Material**

**PlasticDB: a database of microorganisms and proteins linked to plastic biodegradation**

Victor Gambarini^1^*, Olga Pantos^2^, Joanne M. Kingsbury^2^, Louise Weaver^2^, Kim M. Handley^1^ and Gavin Lear^1^

^1^School of Biological Sciences, University of Auckland, 3a Symonds
Street, Auckland 1010, New Zealand.

^2^The Institute of Environmental Science and Research, 27 Creyke Road, Ilam, Christchurch 8041, New Zealand.

* Corresponding author.

victor.gambarini@gmail.com

**Supplementary Table 1.** Example output from the Annotate Gene tool showing the hits for putative plastic-degrading proteins. The input data was a cutinase from *Fusarium verticillioides*.

| Query Sequence | DB Hit ID Number | Percent Identity | E-value | Enzyme Type | Species | Plastic | Secreted |
| --- | --- | --- | --- | --- | --- | --- | --- |
| 00009 | 00009 | 100.0 | 1.5e-168 | Cutinase | Fusarium verticillioides | PCL | Yes |
| 00009 | 00008 | 98.7 | 1.6e-165 | Cutinase | Fusarium verticillioides | PCL | Yes |
| 00009 | 00092 | 93.5 | 1.6e-135 | Cutinase | Fusarium solani | PMCL | Yes |
| 00009 | 00075 | 76.7 | 1.3e-115 | Cutinase | Fusarium oxysporum | PCL | Yes |
| 00009 | 00075 | 76.7 | 1.3e-115 | Cutinase | Fusarium oxysporum | PET | Yes |


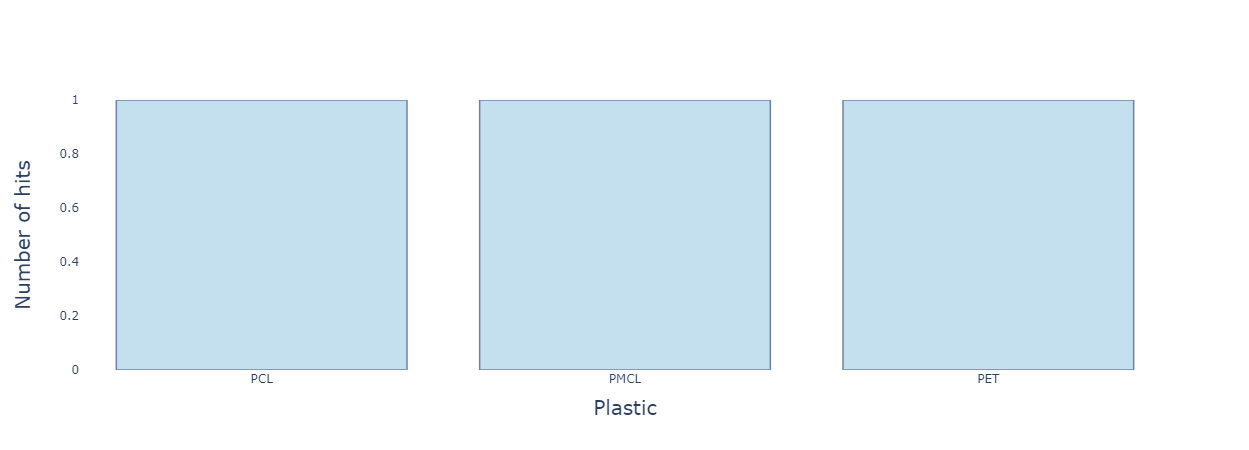


**Supplementary Figure 1.** Example graph output from the Annotate Gene tool. It plots the number of hits (which means the number of proteins in the inputted genome similar to proteins proposed to degrade a specific plastic) per plastic-type. The input data was a cutinase from *Fusarium verticillioides*.
